# Relevant treatment outcomes for individuals aged 60 and older with massive rotator cuff tears: a qualitative study with 16 patients

**DOI:** 10.2340/17453674.2025.43474

**Published:** 2025-04-14

**Authors:** Cristina BARRUFET, Víctor ZAMORA, Catalina LIZANO-BARRANTES, Carlos TORRENS, Andrea BURÓN, Emilio CALVO, Lluis PEIDRÓ, Joan MIQUEL, Raúl BARCO, Montse FERRER

**Affiliations:** 1 Epidemiology and Evaluation Research Group, Hospital del Mar Research Institute, Barcelona, Spain; 2 Department of Medicine and Life Sciences, Universitat Pompeu Fabra, Barcelona, Spain; 3 RICAPPS (Network for Research on Chronicity, Primary Care, and Health Promotion), Madrid, Spain; 4 Health Services Research Group, Hospital del Mar Research Institute, Barcelona, Spain; 5 CIBER en Epidemiología y Salud Pública, CIBERESP, ISCIII, Spain; 6 Department of Pharmaceutical Care and Clinical Pharmacy, Faculty of Pharmacy, Universidad de Costa Rica, San Jose, Costa Rica; 7 Department of Orthopedics, Hospital del Mar, Barcelona, Spain; 8 Department of Orthopaedic Surgery and Traumatology, Hospital Universitario Fundación Jiménez Díaz, Universidad Autónoma de Madrid, Madrid, Spain; 9 Orthopaedics and Trauma Department, Hospital Clínic de Barcelona, Barcelona, Spain; 10 Orthopaedics and Trauma Department, Parc Taulí Hospital Universitari, Institut d’Investigació i Innovació Parc Taulí (I3PT-CERCA), Universitat Autònoma de Barcelona, Sabadell, Spain; 11 Department of Orthopedic Surgery, Hospital Universitario La Paz, Madrid, Spain

## Abstract

**Background and purpose:**

Qualitative research on individuals with massive rotator cuff tears (MRCT) is scarce. This study aims to identify the perceptions, concerns, and treatment outcomes relevant to individuals with MRCT of the shoulder, as expected before treatment or experienced afterward.

**Methods:**

A qualitative study was designed using a hermeneutic phenomenological methodology. Purposive sampling was employed to identify potentially eligible patients (diagnosed with MRCT and aged 60–85 years) in the trauma and orthopedic outpatient clinics of 3 hospitals in Spain. The study employed 2 segmentation criteria: type of treatment and timing (before/after treatment). 16 interviews were conducted to capture the patients’ perspective: 9 were semi-structured and 7 were in-depth. Interpretative phenomenological analysis was used, and triangulation was performed by 3 researchers with diverse backgrounds.

**Results:**

The mean age of participants (10 women and 6 men) was 71 years, with most having their dominant limb affected. The analysis revealed 4 themes (13 subthemes): MRCT impact on daily living (shoulder-derived functional limitation, pain, and emotional disturbance); treatment outcomes (recovering independence, pain relief, and social participation); clinical management (communication with health professionals, duration of the diagnostic/therapeutic process, and participation in decision-making); and characteristics of the individual (sex, work, and comorbidity). Special unmet needs were identified for women, with more prolonged diagnostic and therapeutic processes while bearing most household responsibilities.

**Conclusion:**

Recovering independence and pain relief were the principal outcomes from the perspective of patients with MRCT, and social participation and emotional well-being were closely linked. Measuring these outcomes could improve shared decision-making, while addressing systemic barriers to enhance patient participation.

Rotator cuff tears affect approximately 22% of the population aged 65 and older [1]. Severe cases, defined as massive rotator cuff tears (MRCT), represent from 10% to 40% of these [2,3]. In most cases, non-surgical treatment is the first choice; however, surgery may be necessary if this proves ineffective [4]. Surgery for rotator cuff tears and MRCT is increasing [5,6], with arthroscopic surgery and reverse total shoulder arthroplasty as primary approaches [4].

Patient-reported outcome measures (PROMs) have been incorporated into shoulder treatment effectiveness evaluations to complement clinical assessments [7,8]. A systematic review identified 32 shoulder-specific PROMs [9], none of which were specifically designed for patients with MRCT, and only 2 were validated in samples of patients with full-thickness tears of varying sizes, massive (> 5 cm) or smaller tears: the Rotator Cuff Quality of Life (RC-QOL) [10] and the Short Western Ontario Rotator Cuff Index (SHORT-WORC) [11]. Moreover, shoulder-specific PROMs exhibited substantial inconsistencies in the dimensions they covered, without reflecting patient-specific factors such as sex [12].

To address the existing gap in knowledge regarding the experiences, symptoms, and impacts on the daily life of individuals with shoulder disorders, the Outcome Measures in Rheumatology (OMERACT) Shoulder Core Set Working Group conducted a narrative synthesis of qualitative studies [13], which led to the development of a conceptual model. Few qualitative studies [14,15] identified in this review specifically focused on patients with rotator cuff-related conditions: 1 on patients with full-thickness degenerative tears without trauma [15], and the other on volunteers recruited from the local community with at least 3 months of shoulder pain and a largely preserved range of motion [14] .

Further qualitative research focusing on individuals with MRCT is essential to understand the perceptions, beliefs, needs, concerns, and treatment outcomes that patients with this most severe stage of the condition consider relevant. The identification of outcomes that are truly relevant from the perspective of those who are affected can facilitate their communication with clinicians when selecting treatment, increasing active patient participation in shared decision-making [16], which draws on and deepens in the principles of patient-centered care.

This study aims to identify the perceptions, concerns, and treatment outcomes relevant to individuals with MRCT of the shoulder, as expected before treatment or experienced afterwards. 

## Methods

### Study design

This study employed a qualitative hermeneutic phenomenological design, appropriate for the in-depth exploration of patients’ perceptions of their condition and outcomes [17], applying an interpretative strategy. It was the first qualitative phase of a mixed methodology project with an exploratory sequential design variant [18] to develop variables (outcomes perceived as relevant to individuals with MRCT) and assess the effectiveness of treatments from the patients’ perspective in a final quantitative test. The study was reported following the COnsolidated criteria for REporting Qualitative research (COREQ) [19] (checklist in Supplementary Table 1).

### Participants

Potentially eligible patients with an MRCT were identified in the trauma and orthopedic outpatient clinics of 3 hospitals in Spain—2 public tertiary centers and 1 private one. Their orthopedic surgeon initially invited them to participate in the study, and they were contacted afterwards via telephone by the research team. Written informed consent was obtained from all participants prior to the interview.

The inclusion criteria for this study were patients aged 60 to 85 years, diagnosed with an MRCT (> 5 cm) through magnetic resonance imaging, regardless of the presence of arthritis. The exclusion criteria included evidence of glenohumeral instability managed conservatively, active shoulder infection, previous fracture, surgery, or neurological injury affecting the shoulder, as well as any condition that could hinder oral communication.

To ensure a diverse range of perspectives and promote heterogeneity in conceptual meanings, purposive sampling was employed with 2 segmentation criteria: type of treatment (physical therapy, arthroscopic surgery, or prosthetics) and timing of the assessment (before or after treatment). To avoid responses during the acute postoperative period and to capture topics that persist over time, participants were interviewed between 3 months and 2 years after treatment. Additionally, a maximum variation sampling method was used, based on the following variables: age, sex, affected arm, household members, and educational level.

### Sample size

Sample size was determined based on the concept of information power [20], which places emphasis on the adequacy, quality, and variability of the data collected rather than the number of participants. Recruitment ceased when researchers concluded that the data collected was varied, relevant, and sufficiently detailed to address the study’s aim.

### Interviews

The first author conducted audio-recorded, semi-structured individual interviews using a pre-established guide encompassing 5 topics related to shoulder injury: (i) physical manifestations and daily life activities, (ii) quality of life, (iii) social participation and leisure activities, (iv) needs, concerns and treatment, and (v) expectations for treatment. The guide was developed by the research team to encourage participants to elaborate on their experiences while allowing flexibility to explore emergent themes. This guide included open-ended questions and prompts based on the conceptual model of the OMERACT Shoulder Core Set Working Group [7,13,21] and core domains from certain PROMs [22–24]. After conducting and analyzing 9 interviews, the data collected on the first 3 topics was sufficiently varied and detailed, whereas data on the last 2 topics did not hold enough detail. To adequately address the study aims, we decided to transition from semi-structured to in-depth interviews, focusing on the topics that needed further exploration: (a) from injury to treatment experience, (b) the shared decision-making process, and (c) treatment outcomes. Both the initial and the modified versions of the guide are presented in Table 1.

**Table 1:** Guide for patients’ interviews

**Introduction (“setting the scene”, creating a relaxed atmosphere):**
Presentation of the interviewers, the purpose of the research project, and the purpose of the interview; and asking for sociodemographic information and clinical characteristics
**First version (semi-structured interviews)**
1. Physical manifestations and daily life activities related to shoulder injury What discomfort does a shoulder injury cause you? Does your shoulder problem affect the activities of your daily life? How important is it to lift weight or move your arm freely? 2. Quality of life related to shoulder injury What is quality of life for you? Does your shoulder problem affect your quality of life? What do you think could improve your quality of life? 3. Social participation and leisure activities Does your shoulder injury prevent you from doing activities that you enjoy and/or are interested in? Has your role within the family changed? Do you see your friends and family as often as you did before your shoulder injury? Do you need help to do the things your shoulder prevents you from doing? Do you have someone to take care of you? Do you have someone in your charge? 4. Needs, concerns, and treatment related to shoulder injury ^a^ What worries you about your shoulder problem? What is your experience with visiting professionals who treat you for your shoulder problem? ^b^ Have the treatment options been explained to you in a comprehensible way? Have you been asked for your opinion? Have you been involved in treatment decisions? Do you have concentration and/or memory problems due to shoulder pain? Do you have little interest in doing things or do you enjoy them less? Do you have someone you can talk to? What helps you feel better? What do you do when you feel worried? 5. Expectations for treatment ^c^ What do you expect from the treatment proposed for your shoulder injury? ^c^ In the case of having undergone any treatment, is there any aspect of the treatment that has gone especially well for you? ^c^ What do you expect from the evolution of your problem in the future? Do you think it could improve your situation?
**Second version (in-depth interviews performed after domiciliary lockdown)**
From injury to treatment experience: circumstances and reasons that led to the proposed treatment; access to treatment through the health system; diagnostic delay Shared decision-making process: who/where/how reports the information; needed information; responsibilities (family caregivers, work); professional–patient relationship; educational level, age, comorbidities Treatment outcomes: how they see themselves after treatment; expected and experienced improvements in health status, well-being, and relationships with friends and family

Superscript a, b or c in the semi-structured interviews open-ended questions link with topics deeply explored in subsequent in-depth interviews.

16 interviews were conducted between February 2020 and April 2023, either face-to-face in hospitals or remotely (online or by telephone), according to the different phases of SARS-CoV-2 pandemic restrictions. Most interviews were conducted with only the participant and researchers present. The interviews were conducted in Spanish and/or Catalan, with a median audio recording length of 45 minutes, and field notes were taken. At the end of each interview, the informants were given a verbal summary to confirm that the main ideas had been accurately identified, allowing them to address any aspects with which they disagreed or felt uncomfortable.

### Data analysis, rigor, and quality control

An interpretative phenomenological analysis was used to organize, describe, and interpret the raw data [25], which was selected to systematically explore the phenomenon through an inductive approach and is appropriate for data interpretation from a small sample.

For the analysis, first, the interviews were transcribed verbatim after being de-identified, audio recordings were listened to, and transcripts were read repeatedly until the researcher became familiar with the text. Second, pre-analytical intuitions were formulated after re-reading the transcriptions and field notes. Third, emerging recurring themes derived from the data were identified and coded using Atlas.ti software (Scientific Software Development GmbH, Berlin, Germany). Fourth, data was grouped and illustrated using matrices and charts to identify common patterns, convergences, divergences, and contradictions. Fifth, the process continued with each subsequent interview, maintaining an iteration cadence throughout the process to exchange information between data collection and analysis. Finally, the authors discussed the identified themes and data codes, incorporating a sex perspective.

Semi-structured interviews were repeatedly listened to and coded [26] by 1 researcher (CB). A second interviewer was present during the in-depth interviews (VZ or CL-B). Triangulation of the analysis was performed by these 3 researchers—a nurse, a biologist, and a pharmacist—who had no experience in treating patients with MRCT nor any relationship with the participants prior to the study. Therefore, their preconception of the analytical framework was shaped mainly by the literature review and the patients’ cultural context. Rather than attempting to set aside these preconceptions, the team explicitly acknowledged them by comparing the results of our study with the OMERACT conceptual model for shoulder core domains and critically engaged with them to enrich the interpretative process.

The original codes and verbatim quotations in Spanish were translated into English by a bilingual team member (CL-B).

### Ethics, registration, data sharing plan, funding, use of AI tools, and disclosures

The project was reviewed and approved by the Ethics Committee of the Hospital del Mar (2018/7913/I) and was conducted according to the principles of the Declaration of Helsinki.

Ethical considerations in relation to the Spanish Organic Law on the Protection of Personal Data (Organic Law 3/2018 of December 5) were followed, in compliance with regulations promulgated by the Spanish Agency for Data Protection. Research information was provided prior to the interviews, based on the European Parliament’s Regulation 2016/679 of May 5, 2018. The trial was registered in ClinicalTrials.gov (NCT05780229).

Atlas.ti software is a tool for qualitative data analysis offering a wide array of AI-driven analysis tools. No other AI tools were used.

The institution of several authors has received funding from PI18/00152 (CB, VZ, CT, LP, RB, MF) and Generalitat de Catalunya AGAUR 2021 SGR 00624 (VZ, CL-B, MF). 2 authors have received funding for their work in the form of competitive research grants while participating in the project related to the submitted article: OAICE-85-2019 ((CL-B) and ISCIII-FSE FI19/00229 (VZ). None of these funding bodies pose a conflict of interest in connection with the submitted article. Complete disclosure of interest forms according to ICMJE are available on the article page, doi: 10.2340/17453674.2025.43474

## Results

The characteristics of the 16 informants (mean age 71 years) showed that most had their dominant limb affected and were living with companions. 5 participants presented comorbidities, and only 1 experienced a complication (deltoid hypoesthesia, Table 2).

**Table 2:** Characteristics of participants and non-participants

	Participants (n = 16)	Non-participants (n = 5)
Age, median (range)	71.5 (61–81)	70.5 (65–78)
Sex (Female/male)	10/6	4/1
Selected treatment		
Physical therapy	5	1
Arthroscopic surgery	6	
Prosthesis	5	4
Time of assessment		
Before treatment	6	4
After treatment	10	1
Arm allocation		
Dominant limb	10	
Non-dominant limb	2	
Both	4	
Household members		
Living alone	4	
Living accompanied	12	
Educational level		
Illiterate	1	
Primary	10	
Secondary	4	
University	1	
Period of assessment		
Before lockdown	3	
During lockdown	6	
After lockdown	7	
Comorbidity ^a^	5	
Surgical complications ^a^		
None	15	–
Deltoid hypoesthesia	1	–

a Information on comorbidity and surgical complications was obtained from medical records.

**Figure:**
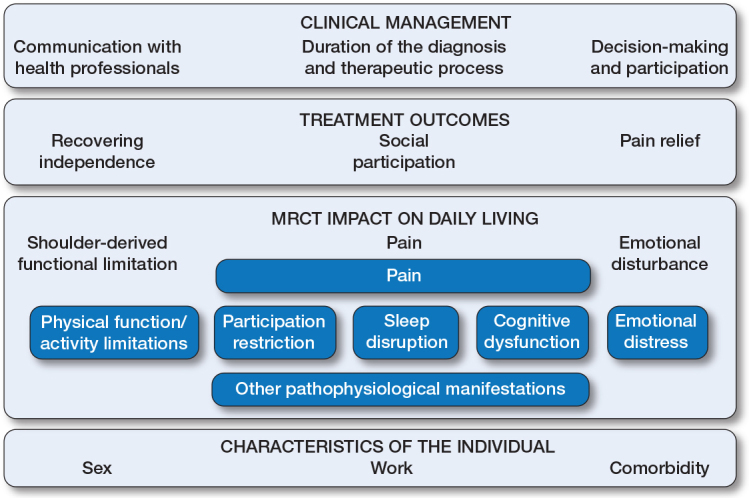
Overview of the results of the thematic analysis within the OMERACT conceptual model, describing the experiences of individuals with shoulder disorders [13]. Dark-blue boxes show the framework developed by OMERACT [13]. Light-blue boxes show the themes and subthemes that emerged from interpretative analysis of the data. The theme “MRCT impact on daily living” aligns closely with several OMERACT core domains: pain, physical function/activity limitations, and emotional distress. 3 further themes—“Treatment outcomes”, “Clinical management”, and “Characteristics of the individual”—fall outside OMERACT core domains but are closely related to them.

The Figure displays in dark-blue boxes the conceptual model developed by the OMERACT Shoulder Core Set Working Group [13], while light-blue boxes show the themes and subthemes that emerged frominterpretative analysis of the data. The final themes, subthemes, and verbatim quotations translated from Spanish are presented in Table 3 (see the original codes and quotations in Spanish in Supplementary Tables 2 and 3).

**Table 3:** Themes and subthemes with verbatim quotations translated into English

**A. Massive rotator cuff tear impact on daily living**
*A.1. Shoulder-derived functional limitation*
“Because I have spent a whole year having to be like this [arm immobilized in a sling] because I couldn’t move my arm” (ES1)
“Until recently my husband bathed me, but now I have said: no! That I want to bathe myself, because I have to see to myself” (ES3)
“I couldn’t put my bra on either, because it was hurting my shoulder. Of course, when I was operated on this one [shoulder] I was braless for a year. I couldn’t fasten it either” (ES4)
“Housekeeping is a day, I am the one who has to get to it. Men aren’t the same. They help in what they can, but it’s not the same…. If my husband isn’t here I’m not going to be waiting for him” (ES3)
“But the thing is that even with the pain, I managed to do everything. No, I didn’t want to resort to anyone because, as I said, my husband would do anything for me, but it wasn’t like that, right? I wasn’t asking anyone for help, but it was painful, but I had to put up with it” (ES5)
“I climb my steps, get my pots out of the cupboards, clean them … a bit, like one of my brothers says, he says I look like a T-Rex dinosaur, because I don’t move my arms away from my body much. But, well, I get by” (ES12)
“I don’t really care for housework” (ES10)
“And, well, [wife] runs the errands and so on, I don’t, you see, she does it all” (ES7)
“I try to never carry, this is, not carry anything on my arm. Not to lift any weights, not to lift anything” (ES2)
“I tried to move my arm as little as possible and that way it even feels better…. I apply some cold, I apply some heat and this way … I keep coping” (ES12)
“Now I’ve got one of these small folding clotheslines, I set it up and hang it. Then I can, right?” (ES3)
“Instead of getting a bottle for watering, I grab a ladle” (ES11)
“Well I leave it, do something else and after a while I go back…” (ES10)
*A.2. Pain*
“I’m going to have to get my arm or my shoulder cut off, I’m going to get something done about the pains that I have and I’ve been going through for a long time, that it wasn’t a day or two, nor a week or two. That it has already been a year” (ES1)
“It all started one day with a bit of pain, I would put some cream on that made it a bit better, I’d take a P, an N … and so on. But now is when … no medication gets rid of it” (ES11)
“uUntil I can’t bear it any more, I’m not an annoying woman who goes to the doctor at the slightest chance, no” (ES4)
“It takes away your concentration because the pain is bad. I mean, you are doing something and then you feel [winces simulating pain], there’s no doubt” (ES2)
“It feels like they stick a stone there and it’s squashing you” (ES11)
“Harmful pain, very aggressive” (ES7)
“Terrible … continuous pain” (ES6)
“I can’t sleep with the pain I have” (ES7)
*A.3. Emotional disturbance*
“Well, you do evolve, I am: why am I going to start walking if I’m always at the same spot?” (ES10)
“Not being able to look after myself. That’s what scares me the most” (ES6)
“Helplessness, that sort of things, that you feel almost incompetent, so as to speak … something useless. Because, when the pain appeared, it would break you” (ES2)
“People are not there to talk about problems, because they all have their own thing going on” (ES3)
“I try to cry at home, where nobody sees me, and when I go out in the street I clean my eyes” (ES4)
“Oh, my dear God, when will I be able to move? When will I be able to do my housework? Even if I don’t do much, but at least I’d be able to move my arm” (ES1)
“I set my mind on something else, because you can’t be always on this. Then was when they took me to this association I go to, because I didn’t have anything else. I’d sit and everything came back to me, and all the things … and that’s it” (ES1)
“If I was feeling a bit down, she’d call me and I’d get to talk with her and forget what I’m having to deal with” (ES1)
“Being able to take care of myself, especially being able to take care of myself, because that’s what scares me most, this thinking that … sometimes … and I’m chaotic, right? But sometimes I say: Oh my God! If I can’t take care of myself, let me be taken, even by the devil, but let me be taken and don’t leave me here if I can’t take care of myself” (ES12)
“I get exasperated. I mean, what am I going outdoors for? What would I do to soothe it?” (ES11)
**B. Treatment outcomes**
*B.1. Recovering independence*
“Well, what I’d like to achieve, it would be my torn right arm to get to do what I used to do just before the tear or what I’m doing with my left arm” (ES10)
“OK, it’s not going to be 100% … but at least that I can get to lift up a glass. Only lift up a glass, a plate from there, and be able to cook my meal, do my own things … even if I can’t reach out to the clotheslines. I’d have that, even if I didn’t achieve anything higher” (ES3)
“But anyway, it’s 71 years, I can’t ask for miracles…. Getting myself dressed on my own, and … correctly! Without doing weird gestures nor strange movements. And being able to wash my hair well, not washing it like this, as I say, with one hand hunched up and the other one. Like, every day, daily things. But that … well you’d like to do them how you used to. That it’s not possible? Well OK…” (ES12)
“I don’t want it to be too fast either, please! If it worsens, well, let it be bit by bit” (ES12)
*B.2. Pain relief*
“That we had an improvement in living with the pain” (ES7)
“I don’t want surgery! I want you to get rid of my pains!” (ES5)
“If they could just take my pain away if I had surgery! Maybe I wouldn’t be like that, maybe I’d be more agile, better … about myself. That’s enough, because to me the pain seems to get into my head and I’m thinking, going over and over the same thing … and if I have to hold hands with one of my daughters or a family member...” (ES11)
“Because of course, having so many pains, I say, I’m going to become disabled in this arm because I can’t do anything. I mean, not for anything else, what I wanted was to get rid of my pains … I don’t want from 1 to 10 or anything like that, doctor, what I want is to get rid of the pains. When he saw me, he said: ‘you need surgery’, and I said: ‘well the sooner, the better’” (ES5)
“Getting rid of the pain and another bit is that I live on my own…” (ES12)
*B.3. Social participation*
“I would go back to it, willingly, because I really like to talk, to chat with people, to ask them things and talk to them, the family. And now we say goodbye or I say goodbye and I don’t feel like stopping and chatting or going out to the market, that I used to go almost every other day, or on Fridays when they set up the street market. Not even that. I don’t feel like it” (ES11)
“Spending more time with them [family] … it’s as if my wings had been clipped so that I can’t fly” (ES11)
“Now I won’t be able to paint for a while, but when I get better I will go [to art classes], and to do my things, go out with my friends, I don’t know, shopping, that I don’t shop, they bring my goods home, but I’d order it. Just going for a walk around there, then here, then over there. To entertain myself a bit. And I’m not going to do much at home either … being home, I have someone who helps me when I have to paint or something, go out with my friends, a weekend here and there, and a drink somewhere to have some fun. That’s all, I don’t want anything more” (ES4)
**C. Clinical management**
*C.1. Communication with health professionals*
“I’m not interested in anything but the technical part. But to know, a photograph of the human skeleton where you can see all the muscles, that shows what they are going to be touching: ‘we removed all of this and we put it there’” (ES2)
“The surgery was before, he [the specialist] gave me the information afterwards” (ES3)
“It seems like the doctor didn’t put there anything about me going to physical therapy, but I consider that, if I’m going, although he spoke about something else…. I made him see that this was good for me, what do you call it, the electric currents, and the infrared lamps, because I can do the exercises at home, but I don’t have these things at home and it was this that I insisted on. But, according to the girl who is doing my physical therapy, my doctor didn’t say anything about me going there” (ES2)
*C.2. Duration of diagnosis and therapeutic process*
“How was I going to be like this for so many years, for so long? … I have no way of asking for help anywhere” (ES11)
“That a family doctor can’t see you … you just say: ‘I’m going to call to check. It’s just … it’s just … the doctor has … of course, I understand, she has a lot of work, it’s over the phone, she doesn’t see you … and of course, what can I say, I get exasperated … when I have to speak twice with that machine, I no longer speak. I hang up and it’s over” (ES12)
“They had me for so long with my tendinitis! That I wish the doctor had taken me and operated on me at that very moment that I suffered so much until they prepared me” (ES1)
“I think that if it were, at least, once a month, maybe I’d feel more relief and I’d feel better” (ES12)
“This has been going on for … not exaggerating, my tendons are torn since ’94! I think that they are torn. And I have gone, but never … as they didn’t send me to do this at the public health system, an MRI, because they don’t do it … they send you to do a, an echography, but you have to see to it. And see, now they have gotten me an MRI, my whole arm was ruined” (ES3)
*C.3. Decision-making participation*
“Not to me, my doctor already said I had to get surgery, that my tendons were torn and that’s it … what else can they say, that’s it. I signed” (ES1)
“They didn’t tell me anything, only that they were torn, that they couldn’t do anything for me because I didn’t even have any muscle mass, and if they operated on me and fitted a prosthesis, it would be useless … explain to me why. Because of course, they only told me this, and then I left terribly upset, and thinking. That’s all we needed” (ES6)
“I understood it. This [arthroscopic decompression] was better than the prosthesis because with the prosthesis, you have to get another one in 10 years’ time” (ES3)
“I’ve got to trust them [specialists], because I don’t understand” (ES3)
“As long as it doesn’t hurt, I’m satisfied … the operation could be a 50 or 60% and I … I’m going to be honest, I totally refused. I said no because of this, I live on my own, and I’m like this, my silly hand, but I can still manage. And the other way, I don’t know whether I’d be able to manage.” (ES12)
“… and if I went through surgery and I got a prosthesis, that it wouldn’t do much. Then we said: ‘well going through surgery for the sake of it, well it’s not worth it’, and that’s how it got settled.” (ES6)
**D. Characteristics of the individual**
*D.1. Sex*
Woman: “I am, so as to say, the engine that moves all the carriages. Then, if I derail, the whole train gets stopped” (ES14)
Woman: “I was doing the tasks, even though I had it bad” (ES11)
Woman: “She, look at this arm, she had it torn because she had it damaged, well she ironed and did everything, even when you’d tell her not to do this because it’s going to harm her” (ES4)
Woman: “Well, well, that I broke my whole hand, and he said: you’re crazy, how are you going to break your hand…!” (ES3)
Man: “I always get told off at home: ‘you haven’t done this, you haven’t done that…’, because I don’t like it, you see the things of … housework, making the beds, dusting…. Those things don’t suit me” (ES10)
Man: “No, usually, as my wife doesn’t work and so on, she does almost all the housework. Now I mop the floor a bit, because, well, to help her a bit. If I need to do something at home, well I also help her, but it’s basically her who is in charge of it all. I do little at home. Well, I’m not used to doing it either” (ES16)
*D.2. Work*
“[I want to] work in the street” (ES2)
“Well, what happened to my shoulder is that … well, from working, it started” (ES11)
“Then I got into cleaning, I got work cleaning. I blamed that, as the mops were very heavy” (ES12)
“Because my arms were very worked on. My arms have been working for many years, because it’s like that, depending on what type of job you have, your arms also get caught” (ES4)
*D.3 Comorbidity*
“It’s all at a time, it hurts, the hernia, when my shoulder goes down ... it’s all. This is the madness that I have, my deformed back. If they look at it, they can’t even see my bones…. This is from working, not from sitting. But hey, that’s life” (ES3)
“I say, better than falling … and when I go out like this with the crutch … if I have to walk a lot or whatever, well I take my crutch” (ES6)
“Well, I already have pains in my bones, my knees, like a person who is already, who’s already old…” (ES 5)

### MRCT impact on daily living

#### Shoulder-derived functional limitation

Individuals interviewed before treatment mainly described functional limitations in raising their arm above their shoulder level, which worsened substantially when pain appeared. These limitations complicate activities of daily life and imply the need for help. Recovering or maintaining the ability to perform self-care tasks was a predominant concern expressed by all informants.

#### Pain

The most frequently reported complaint before treatment was a shattering pain that remained throughout the day (for months and, in some cases, years), needing to go regularly to the emergency room when it became uncontrollable despite oral painkillers. Patients with prostheses or who had undergone arthroscopic decompression expressed substantial pain relief immediately after surgery.

#### Emotional disturbance

All informants referred to concern and fear about the possibility of not recovering independence. Most of them expressed a clear emotional toll from previous conservative treatments that had yielded few benefits. Other important factors included a lack of understanding from their loved ones or the impact of their condition on them, hiding it to avoid feeling like a burden to their families. Willingness to regain mobility and to stop depending on their social environment, particularly their family, was a recurring theme.

### Treatment outcomes

#### Recovering independence

Informants indicated that their expected functional recovery after treatment would be to regain a level of function similar to that before their tear or equivalent to the contralateral shoulder, around 6 months post-intervention or through gradual improvement.

#### Pain relief

Informants expected their level of pain to become tolerable quickly after treatment. Most individuals undergoing surgery expected a substantial reduction or complete elimination of pain.

#### Improvement in social participation

Returning to leisure activities and participating in the community was identified as a crucial aspect of recovery.

### Clinical management

#### Communication with health professionals

Most informants expressed that they would have needed to know the severity of their condition and its implications for daily life from the outset. Some patients mentioned having gone spontaneously to hospital to seek information.

#### Duration of the diagnosis and therapeutic process

Participants widely agreed in finding an excessive delay between the onset of symptoms and the start of diagnosis and treatment, in some cases spanning decades. They primarily attributed this to the underestimation of their pain by health professionals, despite repeated visits to the emergency department.

Those who underwent conservative treatment complained about the short duration and low frequency of physical therapy sessions. They considered home exercises unproductive and that they prolonged the process.

#### Decision-making participation

All narratives indicated a general tendency to accept the treatment recommended by the physician. Informed decision-making emerged predominantly in informants undergoing surgery, who received information concerning their procedure but noted a lack of opportunities to ask further treatment-related questions.

### Characteristics of the individual

#### Sex

Informants of both sexes explained that household and family care activities are considered the responsibility of the female family members. As men do not consider themselves responsible for these activities, this might lower their own perception of impairment when compared with the women. Conversely, some female informants stated that male family members tended to dismiss or downplay the severity of their injury, believing they were exaggerating.

#### Work

Most informants attributed MRCT to work-related tasks involving repetitive movements and traumatisms. However, those of working age expressed the need to regain function to return to their previous position.

#### Comorbidity

Informants spoke of the effect of comorbidities, particularly those affecting the musculoskeletal system, on the exacerbation of MRCT impact. No further differences were identified based on age or overall health status.  

## Discussion

We aimed to identify the perceptions, concerns, and treatment outcomes relevant to individuals with MRCT of the shoulder, as expected before treatment or experienced afterwards in a qualitative study design.

Our findings contribute to gaining understanding of the impact of MRCT on the daily living, therapeutic process, and treatment outcomes relevant to patients with this condition. Recovering independence and pain relief emerged as the principal outcomes from the perspective of patients with MRCT. Other outcomes, such as social participation and emotional well-being, were also found to be relevant and closely related to both principal outcomes. Our results suggest that individuals with this condition face a complex, delayed, and prolonged diagnostic and therapeutic process. The main systemic barrier identified was the lack of accessibility to health professionals for obtaining additional information or addressing concerns. Informed decision-making emerged predominantly in informants undergoing surgery; however, the tendency was mainly to accept the treatment recommended by the physician. Sex, work, and comorbidities constitute relevant individual characteristics.

Our informants clearly emphasized that functional limitations and pain are both major drivers in the experience of the MRCT condition and therapeutic process. Recovering independence—i.e., avoiding the need for support in daily life or returning home after having to move to a relative’s household—is the most relevant outcome for individuals with MRCT. The perception of being dependent leads to a deep emotional disturbance and a decline in their perceived health-related quality of life. A systematic review [27] of clinical trials in patients undergoing arthroscopic repair of MRCT highlighted the limited comparability of activities of daily life due to inconsistencies in their measurement. Nevertheless, the number and type of daily life activities limited by their condition are not relevant outcomes for individuals with MRCT; their real focus is on the dependence they suffer. Although shoulder-specific PROMs assess activities of daily life, neither these instruments nor previous studies address independence/dependence in terms of the need for help as an important phenomenon.

Our findings, which indicate that pain is a major driver in the experience of MRCT, align with previous studies on individuals with rotator cuff disease [28,29]. However, neither of the 2 PROMs validated in samples including patients with MRCT [10,11] contains a pain dimension, nor do half of the shoulder-specific PROMs identified in a systematic review [9]. Pain is a key dimension to cover when measuring the MRCT impact on patients or assessing the effectiveness of treatment options. No standard set for patients with MRCT has been developed by the International Consortium for Health Outcomes Measurement (ICHOM), while the Core Outcome Measures in Effectiveness Trials (COMET) initiative includes a proposal, although it is specifically focused on arthroscopic rotator cuff repair [30].

Participating in social life also emerged as a crucial outcome in our study interviews. Restricted participation, including work disruption, limited recreational activities, and reduced social interactions, had also been described in previous studies [12,14,15,31]. However, a systematic review of outcomes reported in clinical trials for shoulder disorders found that fewer than 10% had assessed work participation [7]. Furthermore, among the more than 30 existing shoulder-specific PROMs, only 6 contain dimensions related to work and recreational or athletic activities [8,9]: the Shoulder Rating Questionnaire (SRQ), the Western Ontario Rotator Cuff Index (WOSI), the Melbourne Instability Shoulder Scale (MISS), the Munich Shoulder Questionnaire (MSQ), the Oxford Instability Score (OIS), and the Rotator Cuff Quality of Life (RC-QOL). In addition, the Disabilities of the Arm, Shoulder, and Hand (DASH) questionnaire [32], developed for patients with upper extremity musculoskeletal conditions, also covers their impact on occupation within the social dimension. Clearly, all these aspects that conform to the participation of individuals in society have been neglected in the assessment of patients with MRCT. Addressing them in future research could lead to a more comprehensive evaluation of patient outcomes.

There are notable differences between men’s and women’s attitudes towards and access to healthcare. Women expressed dealing with an erratic and prolonged diagnostic and therapeutic process while also bearing the majority of household responsibilities. Some women also felt that their condition was minimized by their relatives. A few studies provided insights into the relationship between sex and rotator cuff pathology, identifying a pattern of poorer outcomes in female patients, which is consistent with our findings [33-35]. Sex-related differences in lifestyle scores on the Western Ontario Rotator Cuff Index (WOSI) before surgery were found in 2 studies [34,35]. Additionally, a prospective cohort [33] showed greater early pain and poorer function in females, compared with males, until 3 months post-surgery, measured with the American Shoulder and Elbow Surgeons questionnaire (ASES).

Our study identifies 2 main barriers that hinder the shared decision-making process: insufficient information and limited access to health professionals for further clarification or to comment on doubts. Moreover, a tendency was found to accept the treatment recommended by the physician, regardless of the amount of information received. In line with the principles of patient-centered care, identifying outcomes that are relevant to patients and ensuring they receive adequate information on these outcomes could serve as major facilitators during the MRCT treatment process. This approach may also enhance patients’ active participation in shared decision-making.

### Strengths

The applied procedural rigor—including verification of information accuracy after each interview, triangulation by researchers with diverse health sciences backgrounds and qualitative methods knowledge, independent double analyses of sexist discourses, the interdisciplinary research team’s reflexivity, and the clear presentation of major results within 4 themes and 13 subthemes—ensures the findings’ consistency with data, validity, and reliability. Additionally, the presence of 2 researchers in the in-depth interviews can enrich data collection by introducing diversity in interviewer characteristics and promoting greater emotional comfort [36,37]. On the other hand, some informants were severe cases (i.e., both shoulders affected or having undergone different treatment strategies throughout their therapeutic process), which had been considered as an exclusion criterion in the previous qualitative study on individuals with full-thickness degenerative tears [15].

### Limitations

Our results may reflect the experiences and perspectives of individuals who are more involved and proactive in their therapeutic process. Furthermore, restrictions imposed during the SARS-CoV-2 pandemic also could bias the informants’ profiles: those individuals with more comorbidities or caregiving responsibilities were reluctant to attend face-to-face interviews, persons living in nursing homes could not participate, and identifying and contacting new participants proved more challenging. Nevertheless, the shift to online or telephone interviews, along with the extension of the study period, enabled the inclusion of a diverse range of perspectives, the fulfilment of the 2 planned segmentation criteria (treatment type and timing of assessment) and heterogeneity in age, sex, affected arm, comorbidity, household members, and educational level. Finally, our pre-understanding has an influence on the analysis process and consequently the findings, but we have made an effort to explicate them clearly.

### Conclusions

Recovering independence and pain relief emerged as the principal outcomes from the perspective of patients with MRCT, closely linked to social participation and emotional well-being. Measuring these outcomes could improve shared decision-making while addressing systemic barriers to enhance patient participation throughout the diagnostic and therapeutic process. Additionally, specific needs of women and working patients should be considered.

### Supplementary data

Supplementary data with Supplementary Table 1 (COnsolidated criteria for Reporting Qualitative research [COREQ] checklist), Supplementary Table 2 (Themes, subthemes, coding, and their definitions), and Supplementary Table 3 (Themes with the original verbatim quotations in Spanish) is available on the article page, doi: 10.2340/17453674.2025.43474

## Supplementary Material



## Notes

CT, MF, and CB participated in the planning and design of the study. CB, CT, EC, LP, JM, and RB were involved in recruitment and the collection of clinical data. CB conducted all the interviews, and VZ and CL-B attended and assisted with some interviews. CB, VZ, CL-B, and AB analyzed the data and contributed to the interpretation of results. CB and MF wrote the manuscript draft. VZ, CL-B, CT, AB, EC, LP, JM, RB, and MF participated in the critical revision of the manuscript. MF and CT obtained funding through grants. VZ and MF supervised the study. All authors have provided final approval of the version to be published and agree to be accountable for all aspects of the work.

The authors wish to thank the patients who have participated in these interviews. They also want to thank Enriqueta Pujol-Ribera for her guidance in the preparation of the protocol and the interviews, during the first steps of this qualitative study. They thank Áurea Martín for her support in English editing, proofreading, and preparing this manuscript for submission. Finally, they thank the Instituto de Salud Carlos III FEDER (PI18/00152) and Generalitat de Catalunya AGAUR (2021 SGR 00624) for funding this study, the University of Costa Rica OAICE-85-2019 for funding CL-B’s PhD grant, and the Instituto de Salud Carlos III-Fondo Social Europeo (FI19/00229) for funding VZ’s contract. 

Handling co-editors: Jeppe Vejlgaard Rasmussenand Robin Christensen.

*Acta* thanks Charlotte Myhre Jensen, Birgitte Hougs Kjær and other anonymous reviewers for help with peer review of this manuscript.
