# Supplementary material for: Relevant treatment outcomes for individuals aged 60 and older with massive rotator cuff tears: a qualitative study with 16 patients
Source: Acta Orthop. 2025 Apr 14;96:322–30. doi: 10.2340/17453674.2025.43474 (PMC11995431; doi:10.2340/17453674.2025.43474)
Supplement: Supplementary file 1 [file ActaO-96-43474-s1.pdf]

SUPPLEMENTARY TABLE 1. COREQ (COnsolidated criteria for REporting Qualitative research) Checklist

Topic	Item No.	Guide Questions/Description	Reported on Page No.
**Domain 1: Research team and reflexivity**			
Personal characteristics			
Interviewer/facilitator	1	Which author/s conducted the interview or focus group?	Page 8
Credentials	2	What were the researcher's credentials? E.g. PhD, MD	Authors list
Occupation	3	What was their occupation at the time of the study?	Page 9
Sex	4	Was the researcher male or female?	Page 8
Experience and training	5	What experience or training did the researcher have?	Page 9
Relationship with participants			
Relationship established	6	Was a relationship established prior to study commencement?	Page 8
Participant knowledge of the interviewer	7	What did the participants know about the researcher? e.g. personal goals, reasons for doing the research	Page 8
Interviewer characteristics	8	What characteristics were reported about the interviewer /facilitator? e.g. Bias, assumptions, reasons and interests in the research topic	Page 8
**Domain 2: Study design**			
Theoretical framework			
Methodological orientation and Theory	9	What methodological orientation was stated to underpin the study? e.g. grounded theory, discourse analysis, ethnography, phenomenology, content analysis	Page 6
Participant selection			
Sampling	10	How were participants selected? e.g. purposive, convenience, consecutive, snowball	Page 6
Method of approach	11	How were participants approached? e.g. face-to-face, telephone, mail, email	Page 7
Sample size	12	How many participants were in the study?	Page 7
Non-participation	13	How many people refused to participate or dropped out? Reasons?	Page 9
Setting			
Setting of data collection	14	Where was the data collected? e.g. home, clinic, workplace	Page 7
Presence of non-participants	15	Was anyone else present besides the participants and researchers?	Page 7
Description of sample	16	What are the important characteristics of the sample? e.g. demographic data, date.	Page 9

Data collection			
Interview guide	17	Were questions, prompts, guides provided by the authors? Was it pilot tested?	Table 1
Repeat interviews	18	Were repeat inter views carried out? If yes, how many?	Not applicable
Audio/visual recording	19	Did the research use audio or visual recording to collect the data?	Page 8
Field notes	20	Were field notes made during and/or after the interview or focus group?	Page 8
Duration	21	What was the duration of the inter views or focus group?	Page 8
Data saturation	22	Was data saturation discussed?	Page 7
Transcripts returned	23	Were transcripts returned to participants for comment and/or correction?	Page 8
**Domain 3: analysis and findings**			
Data analysis			
Number of data coders	24	How many data coders coded the data?	Page 9
Description of the coding tree	25	Did authors provide a description of the coding tree?	Page 9
Derivation of themes	26	Were themes identified in advance or derived from the data?	Page 8
Software	27	What software, if applicable, was used to manage the data?	Page 8
Participant checking	28	Did participants provide feedback on the findings?	Page 8
Reporting			
Quotations presented	29	Were participant quotations presented to illustrate the themes/findings?<br>Was each quotation identified? e.g. participant number	Page 10-20
Data and findings consistent	30	Was there consistency between the data presented and the findings?	Page 23
Clarity of major themes	31	Were major themes clearly presented in the findings?	Page 23
Clarity of minor themes	32	Is there a description of diverse cases or discussion of minor themes?	Page 10-20

Developed from: Tong A, Sainsbury P, Craig J. Consolidated criteria for reporting qualitative research (COREQ): a 32-item checklist for interviews and focus groups. *International Journal for Quality in Health Care*. 2007. Volume 19, Number 6: pp. 349 – 357

SUPPLEMENTARY TABLE 2. Categories, subcategories, coding and their definitions

**CATEGORY A. MASSIVE ROTATOR CUFF TEAR IMPACT UPON LIFE**	
**Subcategory A.1. Shoulder-derived disability and functional limitation**	
**Code A.1.1.** Dependency, functional limitation pre-treatment<br>**Code A.1.2.** Dependency, functional limitation post treatment	Description of those activities and actions that cannot be fulfilled by themselves because of their shoulder condition (extent of the limitation, rational, examples).
**Subcategory A.2. Pain**	
**Code A.2.1.** Physical pain related to the condition	Level of pain and its characteristics, currently or in the near past.
**Code A.2.2.** Sleep disruption	Description of the massive rotator cuff tear impact upon sleep habits.
**Code A.2.3.** Cognitive impairment	How the massive rotator cuff tear affects their cognitive functioning (trouble remembering, learning new things, concentrating, making new decisions…).
**Subcategory A.3. Emotional Disturbance**	
**Code A.3.1.** Mental health	The perception of how the massive rotator cuff tear has modified their mental health (positively or negatively perceived impact).
**Code A.3.2.** Reported quality of life	Self-perceived quality of life in the moment of the interview.
**Code A.3.3.** Self-perception and health status	How they feel and believe about their health status and image during the interview.
**Subcategory A.4. Coping mechanisms**	
**Code A.4.1.** Inner coping mechanisms	Those resilience mechanisms that depend on oneself.
**Code A.4.2.** Coping skills related to social network, community mechanisms	Those resilience mechanisms that originate or converge between the individual and the community.
**CATEGORY B. TREATMENT EXPECTATION OUTCOMES**	
**Subcategory B.1. Recovering independence**	
**Code B.1.1.** Activities of daily living post treatment	The activities they hope to be able to carry out in the future on a day-to-day basis after the treatment (also, if they feel the same or worse), which ones, to what extent.
**Code B.1.2.** Post treatment improvement	How, when, in what way and to what extent they intend to improve with the treatment.
**Code B.1.3.** Mental health improvement	How they expect that the treatment for the massive rotator cuff tear will affect their mental health (positive or negative impact).
**Subcategory B.2. Pain relief**	
**Code B.2.1.** Pain degree	How they think their level of pain will be in the future, in the mid/long term.
**Code B.2.2.** Impairment avoidance	Referring specifically to all those actions or decisions done to avoid being worse in the mid/ long-term future.
**Subcategory B.3 Improvement in social participation**	

**Code B.2.1.** Community	Characteristics of their expected participation in the community's dynamics' after the treatment (from family to politics).
**Code B.2.2.** Leisure	What activities they expect to be able to carry out after the treatment, without being strictly basic activities of daily living. Also, if they feel the same or worse. It's related both with individual and community level, involving either family and friends (i.e. horticulture, gardening, carpentry). In which way and extent they hope to feel integrated (frequency and reciprocity) in their community after the treatment in a mid/long term (i.e. participation in community meeting centers: market, civic centers, painting workshops, family reunions…).
**Code B.2.3.** Health-related quality of life	How they expect/want to feel and be after the treatment, considering the improvements they expected from the treatment (immediately after the treatment, long-term; i.e. well-being, independence, autonomy).
**CATEGORY C. THERAPEUTIC PROCESS**	
**Subcategory C.1. Communication with health professionals**	
**Code C.1.1.** Patient-professional communication	Opinion about the relationship with the health professionals.
**Code C.1.2.** Shoulder impairment and therapeutic options comprehension	Description of the comprehension of their condition and the understanding of the therapeutic options proposed.
**Code C.1.3.** Treatment	Expectations and preferences in terms of treatment outcomes: functional, emotional and social.
**Subcategory C.2. Duration of the diagnoses and therapeutic process**	
**Code C.2.1.** Interaction with the healthcare system	Opinion about the tempos and frequency or need for a second opinion or tests outside the public system; Helplessness within the system.
**Code C.2.2.** Interaction within the healthcare system	Description of their experience between the injury and the treatment, in terms of service access, treatment opportunities and distance between their home and the hospital.
**Code C.2.3.** Treatment-related experience	Experience regarding the selected treatment in terms of beliefs, sensory perception and individual or collective values.
**Code C.2.4.** Delays in the therapeutic process	Detailed description of the delays.
**Code C.2.5.** Treatment adherence	Reported degree of treatment compliance.
**Code C.2.6.** Therapeutic failure	Perceived adverse effects and/or non-improvement derived from the prescribed treatment for the massive rotator cuff tear.
**Subcategory C.3. Decision-making participation**	
**Code C.3.1.** Selected treatment	Opinion about the treatment chosen with the specialist
**Code C.3.2.** Received information, shared decision-making	Expectations about the information resources (format, language, graphs), who is the informant and frequency of the informative intervention during the shared decision-making process.
**Code C.3.3.** Shared decision-making	Description of the shared decision-making process (aid tools, information received).

**Code C.3.4.** Therapeutic preferences	Expectations about how, in terms of proximity, access and speed, they want their therapeutic process to be. Also, the lack of expectation regarding the therapeutic process (i.e. conformism, complications, tempos, treatment expectations).
**CATEGORY D. CONTEXT.**	
**Subcategory D.1. Sex**	
**Code D.1.1** Burden of disease in women	Burden caused by the shoulder condition and situations associated to sex behavior/conditionings.
**Code D.1.2.** Burden of disease in men	
**Subcategory D.2. Relation with work**	
**Code D.2.1.** Disease cause attribution	Rationale of the cause of the shoulder injury from the patient's point of view.
**Code D.2.2.** Work activity post treatment	Expected type of job and professional activity after the treatment.
**Subcategory D.3. Comorbidities of the musculoskeletal system**	
**Code D.3.1.** Comorbidities	Other pathologies or conditions of the musculoskeletal system

SUPPLEMENTARY TABLE 3. Categories with the original verbatim quotations in Spanish

A. MASSIVE ROTATOR CUFF TEAR IMPACT UPON LIFE
**A.1. Shoulder-derived disability and functional limitation**
"porque yo he estado un año que tenía que estar así [brazo inmóvil en cabestrillo] porque no podía mover el brazo" (ES1)
"Hasta hace poco me ha bañado mi marido, pero ahora le he dicho: ¡no! Que yo me quiero bañar, porque yo tengo que hacer mis cosas" (ES3)
"el sujetador tampoco me lo podía poner, porque me hacía daño en el hombro. Claro, cuando me operé de este [hombro] me estuve un año sin sostén. Tampoco me lo podía abrochar" (ES4)
"La casa es un día, yo soy la que tiene que levantar. Los hombres no son iguales. Te ayudan en lo que pueden, pero no es lo mismo […] Si no está mi marido no voy a estar esperándolo" (ES3)
"pero es que aún con dolores llegué a hacer todo. No, yo no quise echar mano de nadie porque ya te digo, cualquier cosa mi marido me lo hacía, ¡pero que tampoco te creas eh!, que no pedía ayuda a nadie, pero es que me dolía, pero me tenía que aguantar" (ES5)
"Yo me subo a mi escalerita, me saco mis cacharros de los armarios, los limpio…un poco, como dice un hermano, que dice que parezco el dinosaurio Rex, porque no separo mucho los brazos del cuerpo. Pero oye, yo me voy defendiendo" (ES12)
"a mí las faenas de casa no me van mucho" (ES10)
"Y Bueno, [esposa] hace los recados y eso, yo no hago por eso, lo hace todo ella" (ES7)
**A.2. Pain**
"A mí me van a tener que cortar el brazo o el hombro, me van a tener que hacer algo de los dolores que tengo y que llevo ya mucho tiempo, que no era ni un día ni dos, ni una semana ni dos. Que eso era ya un año" (ES1)
"Empezó un día que me duele un poquito, me echaba una crema que me aliviaba un poquito, me tomaba un P, un N…y así. Pero ya ahora llega la hora…que ningún medicamento me lo quita" (ES11)
"hasta que ya no puedo más, no soy una mujer chinchosa que a la mínima voy al médico, no" (ES4)
"concentración te quita porque el dolor es fuerte. Es decir, estás haciendo algo y cuando sentís [se contrae simulando dolor], eso no hay ninguna duda (ES2)
"parece que echen una piedra ahí y te está aplastando" (ES11)
"dolor dañino, muy agresivo" (ES7)
"dolor terrible […] continuo" (ES6)
"del dolor que tengo, no puedo dormir" (ES7)
**A.3. Emotional Disturbance**
"Bueno, si evolucionas, si estoy: ¿para qué voy a ponerme a caminar si estoy siempre en el mismo punto?" (ES10)
"no poder valerme por mi misma. Eso es lo que más miedo me da" (ES6)
"Impotencia, esas cosas, que más casi te sentís inepto como quién dice la palabra… algo inútil. Porque, cuando aparecía el dolor, te hacía quebrar" (ES2)
"La gente no está para contar penas, porque todos tienen lo suyo" (ES3)
"yo procuro llorar en mi casa, que no me vea nadie, y cuando salgo a la calle pues me limpio mis ojos" (ES4)
"ay, Dios mío de mi vida, ¿cuándo podré yo moverme? ¿cuándo podré hacer yo mis faenas?, aunque no haga mucho, pero por lo menos que pueda yo mover el brazo" (ES1)
"valerme por mí misma, sobre todo valerme por mí misma, porque es lo que más miedo me da, eso de pensar que es que…A veces…y soy caótica, ¿eh? Pero hay veces que digo: 'Madre mía, si no me valgo por mí misma, que me lleve, aunque sea el diablo, pero que me lleven y que no me dejen aquí si no me valgo por mí misma" (ES12)
"me desespero. Es decir, ¿para dónde salgo? ¿qué haría para aliviarme?" (ES11)
**A.4. Coping mechanisms**
"trato nunca de llevar, o sea, de no llevar nada al brazo. De no levantar peso, de no levantar nada" (ES2)
"procure mover el brazo lo menos posible y así hasta se me alivia […] me pongo frío, me pongo calor y así…voy tirando" (ES12)

"ahora me he cogido un tendedero de estos plegable pequeñito, lo pongo y me lo voy echando. Entonces voy ¿sabes?" (ES3)
"yo echaba la mente para otro lado, porque decía que no puedes estar entre una cosa de esto. Ya fue cuando me llevaron a la asociación esta de que yo voy, porque no tenía nada más. Me sentaba y me venía todo lo de atrás, y todo lo que…y ya está" (ES1)
"si estaba un poquillo baja, me llama y yo ya me pongo a hablar con ella y se me pasa lo que tengo" (ES1)
"en vez de coger una botella para regar, pues cojo un cacillo" (ES11)
"pues lo dejo, hago otra cosa y al cabo de un rato vuelvo…" (ES10)
**B. TREATMENT EXPECTATION OUTCOMES**
**B.1. Recovering independence**
"Bueno, a mí lo que me gustaría alcanzar, lo que sería roto el brazo derecho por conseguir lo último que yo tenía antes de la ruptura o lo que estoy haciendo con el brazo izquierdo" (ES10)
"Que no me va a quedar el 100%, vale... pero solo que me quede para poder coger un vaso. Solo para coger un vaso, un plato de allí, y poder hacer mi comida, mis cosas…aunque no llegara al tendedero mira. Ya tengo eso, aunque no llegara tan alto" (ES3)
"Y también me gustaría manejar como me manejaba yo antes. Pero hija, vuelvo a las mismas, son 71 años, tampoco puedo pedir milagros […] El vestirme yo sola y… ¡pero bien! Sin hacer gestos raros ni movimientos extraños. Y el poderme lavar la cabeza a gusto, y no que me lavo así la cabeza, como yo digo con la mano encogida y la otra. O sea, cosas que son cotidianas, diarias. Pero que…bueno que te gustaría hacerlas como lo hacías ¿Qué no puede ser? Pues oye…" (ES12)
"tampoco quiero que vaya muy deprisa, ¡por favor! Si va a peor, pues eso, que vaya muy poco a poco" (ES12)
**B.2. Pain relief**
"que tuviéramos una mejoría de vida con el dolor" (ES7)
"¡yo no me quiero operar! Yo quiero que me quites los dolores" (ES5)
"¡Yo con que me quitara el dolor si me operaran! Yo igual no estaba así, igual estaba más ágil, estaba mejor [hace una pausa] conmigo. Eso es lo suficiente, porque a mí el dolor parece que es se mete en la cabeza y estoy pensando, dándole vueltas y vueltas a lo mismo […] y si tengo que darle la mano a alguna de mis hijas o familiar" (ES11)
"Porque claro, al tener tantos dolores, digo, me voy a quedar invalida de este brazo porque no puedo hacer nada. O sea que por otra cosa nada, yo lo que quería era que me quitaran los dolores […] yo no quiero del 1 al 10 ni nada de esto, yo doctor quiero que me quiten los dolores. Cuando me vio ya me dijo: 'hay que operarte', y dije: 'pues cuanto antes mejor'" (ES5)
"Que se me vaya el dolor y otro poco es que yo vivo sola…" (ES12)
**B.3. Improvement in social participation**
"Yo ahora lo recuperaría, pero a gusto, porque yo soy muy habladora, de darme así con la gente, de preguntarle y hablar con ellos, la familia. Y ahora decimos adiós o digo yo adiós, y no tengo ganas de pararme y hablar o salir al mercado, que salía casi, día sí día no, o viernes que ponen mercadillo. Ni eso tampoco. No me animo" (ES11)
"estar más con ellos [familia], […], es como si tuviera yo recortadas las alas para que no vuele" (ES11)
"ahora no podré pintar en un tiempo, pero cuando me ponga bien pues sí que iré [clases de pintura], y a hacer mis cosas, a salir con mis amigas, no sé, ir a comprar, que no compro, me suben la compra, encargarla yo. Pues ir a dar una vuelta por ahí, por allá, para acá. Para distraerme un poco. Y en casa pues no voy a hacer mucha cosa tampoco […] estar en mi casa, tengo una persona que me ayuda y yo cuando tengo que pintar o alguna cosa, salir con mis amigas, un fin de semana para arriba para abajo, y tomando algo por ahí a pasarlo bien. Ya está, no pido más " (ES4)
**C. THERAPEUTIC PROCESS**
**C.1. Communication with health professionals**
"Más allá de lo técnico no me interesa nada. Pero saber, una foto del esqueleto humano donde aparezcan todos los músculos, que te ponga lo que van a tocar: 'sacamos todo lo de acá y lo pusimos allá." (ES2)

"La operación fue antes, él [especialista] me ha dado la información después" (ES3)
"aparentemente el doctor no puso nada ahí de que yo voy a rehabilitación, pero considero que, si yo voy, si bien él habló de una cosa…yo le hice ver de que me hacía muy bien esto, cómo es, las corrientes, y las lámparas de infrarrojo, porque yo los ejercicios pues los hago en casa, pero esto no lo tengo en casa y en eso fue que hice hincapié. Pero, según lo que la chica que me está haciendo rehabilitación, el doctor no puso nada de que yo vaya allí" (ES2)
**C.2. Duration of the diagnoses and therapeutic process**
"¿cómo iba a estar así tantos años, tanto tiempo? […] No tengo manera de pedir auxilio por ningún sitio" (ES11)
"que no te pueda ver un médico de cabecera…es que tú dices: 'voy a llamar para consultar. Es que…es que…es que la doctora tiene…claro, yo lo comprendo, tiene mucho trabajo, es por teléfono, no te ve…y claro, pues qué quiere que le diga, yo me desespero…cuando tengo que hablar dos veces con la maquinita, ya no hablo. Cuelgo y se acabó" (ES12)
"¡me tuvieron tanto tiempo con la tendinitis! Que ojalá me hubiera cogido el doctor y me hubiera operado en aquel mismo momento que tanto sufrí hasta que no me prepararon" (ES1)
"yo creo que, si fuera, por lo menos una vez al mes, a lo mejor sentía más alivio, y me sentía mejor" (ES12)
"esto lleva ya…. ¡sin exagerarte, roto, los tendones desde el 94! Creo yo que están rotos. Y he ido, pero nunca… como no me mandaron hacer esto en la seguridad social, una resonancia, porque no te lo hacen… te mandan una ésta, una ecografía, pero hay que mirarlo. Y ves, ahora me han hecho una resonancia, estaba todo el brazo hecho polvo" (ES3)
**C.3. Decision-making participation**
"A mí no, ya el médico me dijo que había que operarme, que los tendones estaban rotos y ya está…que me van a decir, ya está. Firmé" (ES1)
"no me dijeron nada, simplemente que estaban rotos, que no me podían hacer nada porque incluso no tenía masa muscular, y si me operaban y me ponían una prótesis, que no serviría de nada […] explícame el por qué. Es que claro, solamente me dijo eso y me fui luego deprimidísima, y pensando. Pues sí que estamos bien" (ES6)
"Yo lo entendí. Era mejor esto [descompresión artroscópica] que lo de la prótesis porque la prótesis a los 10 años otra vez hay que poner otra" (ES3)
"Yo tengo que fiarme de ellos [especialistas], porque yo no entiendo" (ES3)
"yo con que no me duela me conformo […] la operación podía ser un 50 o 60% y yo…voy a ser sincera, me negué en rotundo. Dije que no por eso, porque vivo sola, y yo así, mi manita tonta, pero yo me voy apañando. Y de la otra manera, yo no sé si me voy a poder apañar" (ES12)
"…y si me operaban y me ponía una prótesis, que no me serviría de nada. Entonces dijimos: "pues operarme por operarme, pues la verdad es que no", y así quedó la cosa." (ES6)
**D. CONTEXT**
**D.1. Sex**
WOMEN*: "soy, como dijéramos, la máquina que mueve todos los vagones. Entonces, si yo me descarrilo, queda parado todo el resto del tren" (ES14)*
WOMEN: *"Hacía las cosas, aunque lo tenía mal" (ES11)*
WOMEN: *"ella mira, este brazo, que lo tenía roto porque lo tenía fastidiado, pues ella planchaba y hacía de todo, aunque tú le dijeras que no hagas esto porque te va mal" (ES4)*
WOMEN: *"vamos, vamos, que me he roto toda la mano, y me dijo: ¡tú estás toda chalada, te vas a romper la mano…!" (ES3)*
MEN: *"En casa siempre me echa bronca: 'que si no has hecho esto, que si no lo otro…', porque no me va, o sea a mí las cosas de…de casa, el hacer la cama, el quitar el polvo…Esas cosas no van conmigo" (ES10)*
MEN: *"No, normalmente, como mi mujer no trabaja y tal, las labores domésticas las hace casi todas ella. Yo ahora le fregó un poquito, porque bueno, por ayudarla un poquito. Si necesito hacer alguna cosa en casa, pues también la ayudo, pero básicamente es ella la que se encarga de todo. Yo me hago poco en casa. Bueno, tampoco tengo costumbre" (ES16)*
**D.2. Relation with work**
"[quiero] trabajar en la calle" (ES2)

"Pues bueno…lo que me ha pasado en el hombro es que…bueno, de trabajar, empezó" (ES11)
"Entonces me puse de limpieza, a trabajar de la limpieza. Yo lo achaqué a eso, a que las fregonas pesan mucho" (ES12)
"Porque los brazos están muy trabajados. Tienen los brazos muchos años trabajando, por lo mismo, depende del trabajo que tengas, los brazos también se agarran" (ES4)
**D.3. Comorbidities of the musculoskeletal system**
"Se me ha juntado todo, me duele, la hernia, cuando me baja el hombro…está todo. Esto es el desvarío que yo tengo, la espalda deformada. Si me la miran no se me ven ni los huesos […] Esto es de trabajar, no de estar sentada. Pero bueno, así es la vida" (ES3)
"Digo, antes de caerme…y voy cuando salgo y así con la muleta […] si tengo que caminar mucho o lo que sea, pues me llevo la muleta" (ES6)
"Hombre, ya me duelen los huesos, las rodillas, como una persona ya, que es mayor ya…" (ES 5)
